# Supplementary material for: Myosin-binding protein C regulates the sarcomere lattice and stabilizes the OFF states of myosin heads
Source: Nat Commun. 2024 Mar 23;15:2628. doi: 10.1038/s41467-024-46957-7 (PMC10960836; doi:10.1038/s41467-024-46957-7)
Supplement: Supplementary file 1 — Supplementary Information [file 41467_2024_46957_MOESM1_ESM.pdf]

Supplementary Information for

Myosin-binding protein C regulates the sarcomere lattice and stabilizes the OFF states of myosin heads

Anthony L. Hessel[1,2*], Nichlas M. Engels[3], Michel Kuehn[1,2], Devin Nissen[4], Rachel L. Sadler[5], Weikang Ma[4], Thomas C. Irving[4], Wolfgang A. Linke[1], Samantha P. Harris[5*]

[1] Institute of Physiology II, University of Muenster; Muenster, Germany. [2] Accelerated Muscle Biotechnologies Consultants, Boston, MA, USA. [3] Department of Cellular and Molecular Medicine, University of Arizona; Tucson, AZ, USA. [4] BioCAT, Department of Biology, Illinois Institute of Technology; Chicago, IL, USA.
[5] Department of Physiology, University of Arizona, Tucson, AZ, USA.
* Corresponding authors. E-mail: anthony.hessel@uni-muenster.de, samharris@arizona.edu

The PDF file includes:

Supplementary Figures 1-3
Supplementary Tables 1-12

Supplementary Figure

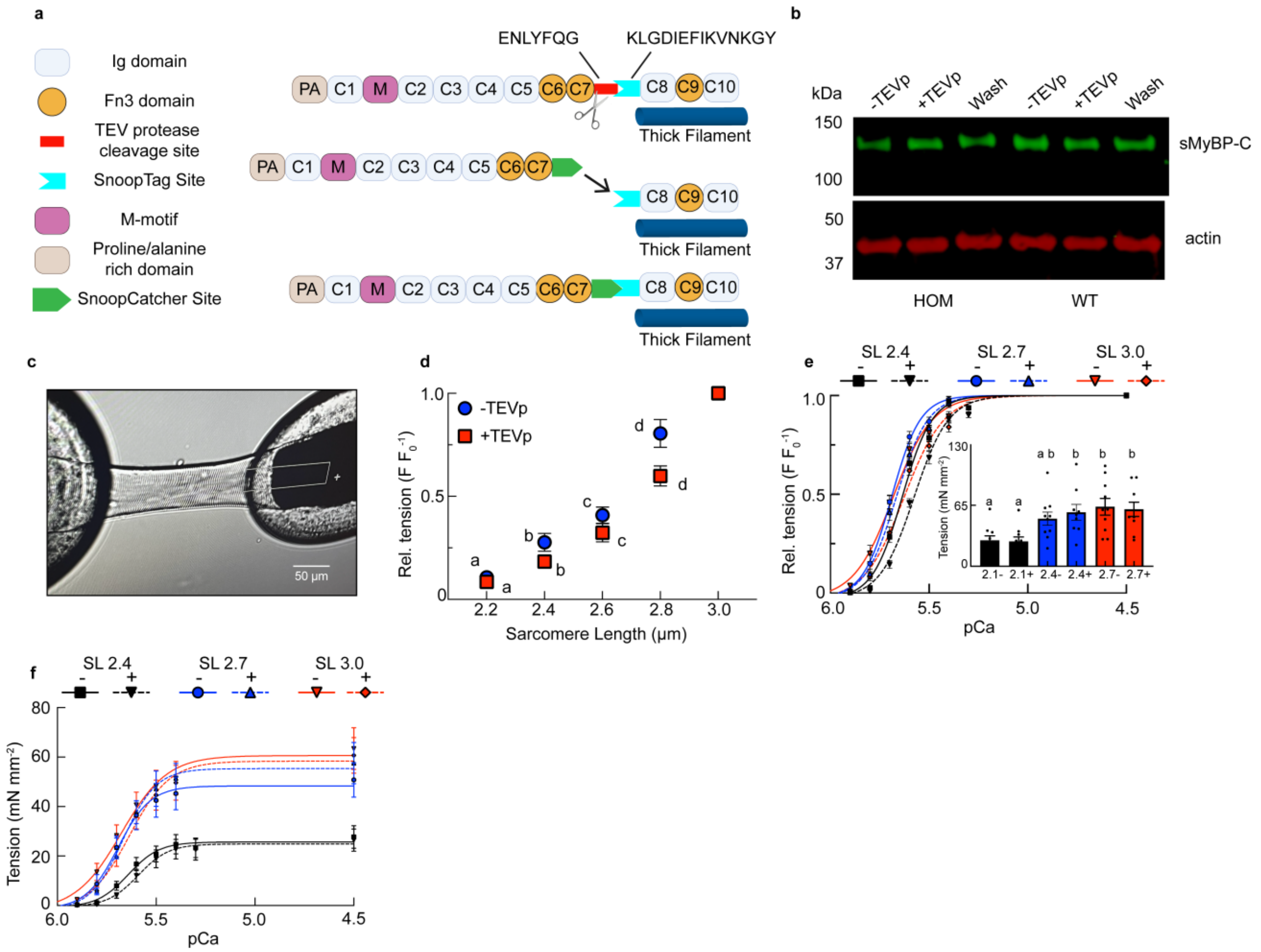

Supplementary Figure 1. **SNOOPC2 mouse line design and evaluation. a** SNOOPC2 mice express a modified fMyBP-C that contains a TEV protease recognition site (red rectangle) and a SnoopTag (cyan trapezoid). The addition of TEV protease cleaves the endogenous C1-C7 domains of fMyBP-C while SnoopTag-C8-C10 remains anchored to the thick filament. Although not employed in this study, it is possible to incubate with a recombinant fMyBP-C construct containing the SnoopCatcher tag (green trapezoid), which will lead to *in situ* replacement of the cleaved fMyBP-C with the recombinant fragment. **b** Western blot of slow MyBP-C paralog from homozygous and wildtype SNOOPC2 psoas, before and after TEV protease treatment. As expected, no cleavage was detected in the slow MyBP-C paralog. This experiment was repeated with similar results three times, two are shown. **c** Representative image of a muscle fiber attachment for a mechanics experiment. The muscle fiber was glued between a motor and force transducer using 100% pure silicone rubber. **d** Passive tension-sarcomere length before (-) and after (+) TEVp treatment, with passive tension normalized to maximal tension at SL 3.0. **e** Tension-pCa relationship at SL 2.4, 2.7, and 3.0 before (-) and after (+) TEVp treatment, with every condition scaled to its maximum (pCa 4.5) tension. **f** Absolute tension-pCa relationship at SL 2.4, 2.7, and 3.0 before (-) and after (+) TEVp treatment. Statistics throughout are repeated-measures ANOVA designs followed by a Tukey Honestly Significant Difference (HSD) post-hoc test on significant main effects. Statistical differences are reported via connecting letters, where conditions assigned to different letters are significantly different. Data throughout reported as mean ± SE. The dataset was generated from n=49 fibers prepared from N=10 mice (6 female / 4 male). Further statistical details are in Supplementary Tables 2, 3, 5-8.

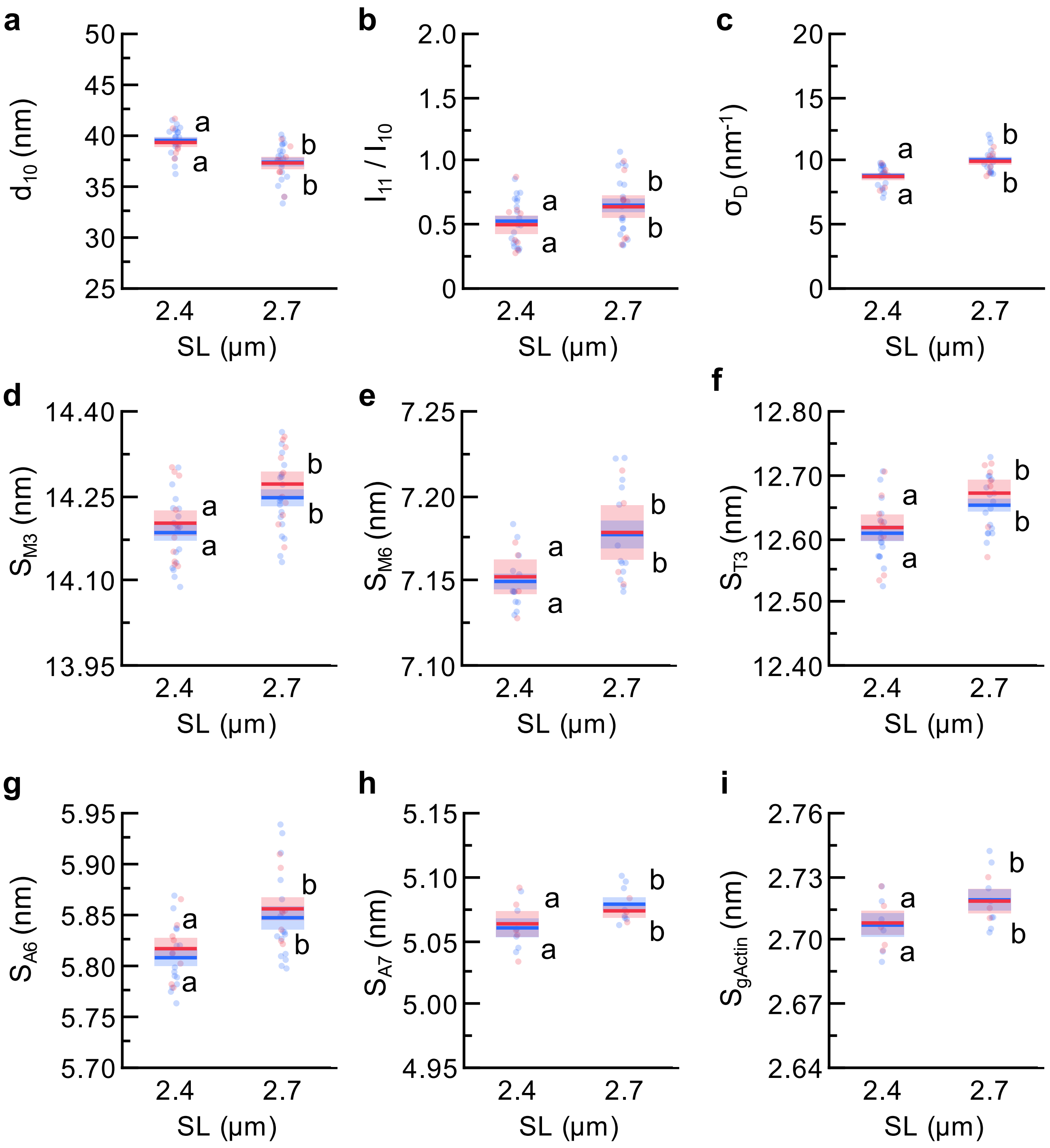

Supplementary Figure 2. **Control dataset for small-angle X-ray diffraction experiments. a-i** X-ray diffraction patterns were collected from wildtype SNOOPC2 psoas fibers under passive conditions at two difference sarcomere lengths (SL), before (blue) and after (red) TEV protease treatment. X-ray features are shown for d_{10} (**a**), I_{11}/I_{10} (**b**), σ_d (**c**), S_{M3} (**d**), S_{M6} (**e**), S_{T3} (**f**), S_{A6} (**g**), S_{A7} (**h**), and S_{gActin} (**i**). As expected, we detected no changes in structural features in wildtype muscles caused by TEV protease treatment. Statistics throughout are ANOVA designs with main effects treatment, SL, and their interaction, and a random effect of individual, followed by a Tukey Honestly Significant Difference (HSD) post-hoc test on significant main effects ($P < 0.05$), and reported in figures as connecting letters: conditions assigned different letters are significantly different. Data throughout reported as mean ± SE. Dataset generated from n=20 fiber bundles prepared from N=6 mice (3 male / 3 female). Full descriptive and statistical details are in Supplementary Table 7.

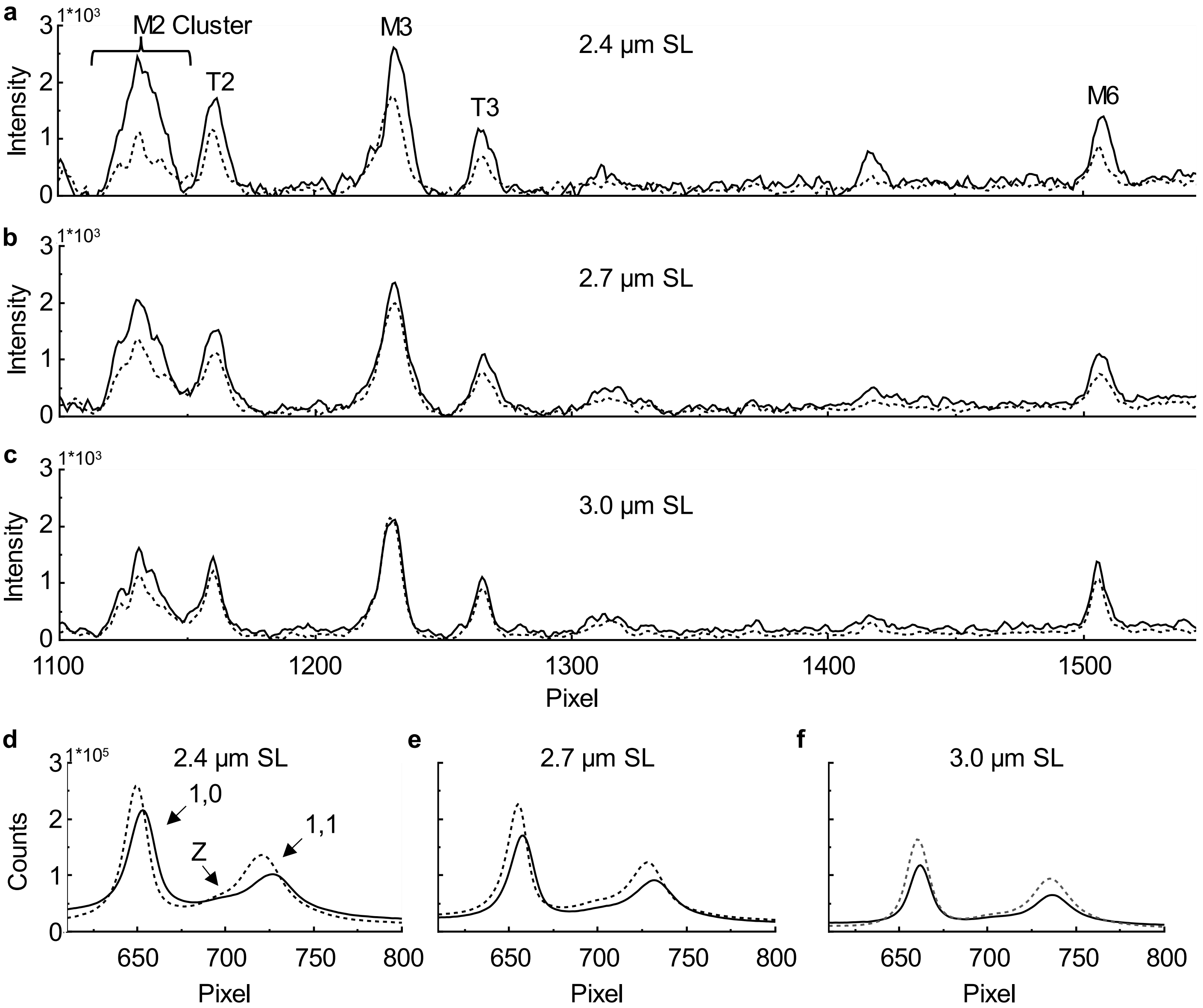

Supplementary Figure 3. **Exemplar 1D intensity profiles of diffraction patterns.** Representative 1D intensity profiles from the meridional (**a-c**) and equatorial (**d-f**) axes, for each sarcomere length (SL), before (solid lines) and after (dashed lines) TEV protease cleavage.

Supplementary Tables

Supplementary Table 1. **Statistical details from experiments shown in Fig. 1g-i. N = number of preparations included in the condition. For SL 2.4, a total of 10 trials were run from psoas collected from 4 mice (2 female / 2 male). For SL 2.7, a total of 10 (9 post) trials were run from psoas collected from 6 mice (4 female / 2 male). For SL 3.0, a total of 10 trial were run from psoas collected from 4 mice (3 female / 1 male).**

Parameter	SL (µm)	Treatment	pCa	N	mean	SE	ANOVA	df 1	df 2	F stat	Prob > F
pCa50	2.4	Pre	4.5-9.0	10	5.61	0.012	SL	2	52	25.33	$\mathbf{2.09x10^{-8}}$
	2.4	Post	4.5-9.0	10	5.55	0.011					
pCa50	2.7	Pre	4.5 - 9.0	10	5.696	0.011	Treatment	1	52	23.00	$\mathbf{1.4x10^{-5}}$
	2.7	Post	4.5 - 9.0	9	5.668	0.009					
pCa50	3.0	Pre	4.5 - 9.0	10	5.683	0.012	Interaction	2	52	0.98	0.38
	3.0	Post	4.5 - 9.0	10	5.627	0.011					

Bold values indicate significant effect. SE = standard error of the mean

Supplementary Table 2. Statistical details from experiments shown in **Fig. 1g-h and Supplementary Fig. 1e. N = number of preparations included in the condition. For SL 2.4, a total of 10 trials were run from psoas collected from 4 mice (2 female / 2 male). For SL 2.7, a total of 10 (9 post) trials were run from psoas collected from 6 mice (4 female / 2 male).**

Parameter	SL (µm)	Treatment	pCa	N	mean	SE
Tension	2.4	Pre	4.5	10	1	0
(mN mm^{-2})	2.4	Pre	5.3	9	0.938	0.03
	2.4	Pre	5.4	10	0.971	0.024
	2.4	Pre	5.5	10	0.786	0.021
	2.4	Pre	5.6	10	0.659	0.03
	2.4	Pre	5.7	10	0.284	0.028
	2.4	Pre	5.8	9	0.026	0.01
	2.4	Pre	5.9	9	0.008	0.004
	2.4	Pre	9.0	10	0	0
	2.4	Post	4.5	10	1	0
	2.4	Post	5.3	9	0.903	0.015
	2.4	Post	5.4	10	0.881	0.02
	2.4	Post	5.5	10	0.685	0.032
	2.4	Post	5.6	10	0.456	0.022
	2.4	Post	5.7	10	0.145	0.02
	2.4	Post	5.8	9	0.01	0.004
	2.4	Post	5.9	9	0.002	0.001
	2.4	Post	9.0	10	0	0
Tension	2.7	Pre	4.5	10	1	0
(mN mm^{-2})	2.7	Pre	5.4	10	0.979	0.017
	2.7	Pre	5.5	10	0.869	0.022
	2.7	Pre	5.6	10	0.791	0.027
	2.7	Pre	5.7	10	0.461	0.034
	2.7	Pre	5.8	10	0.15	0.042
	2.7	Pre	5.9	10	0.005	0.005
	2.7	Pre	9.0	10	0	0
	2.7	Post	4.5	9	1	0
	2.7	Post	5.4	9	0.963	0.024
	2.7	Post	5.5	9	0.835	0.015
	2.7	Post	5.6	9	0.701	0.021
	2.7	Post	5.7	9	0.41	0.042
	2.7	Post	5.8	9	0.097	0.024
	2.7	Post	5.9	9	0.004	0.003
	2.7	Post	9.0	9	0	0

SE = standard error of the mean

Supplementary Table 3. **Statistical details from experiments shown in Fig. 1i and Supplementary Fig. 1e. N = number of preparations included in the condition. For SL 3.0, a total of 10 trial were run from psoas collected from 4 mice (3 female / 1 male).**

Parameter	SL (µm)	Treatment	pCa	N	mean	SE
Tension	3.0	Pre	4.5	10	1	0
(mN mm^{-2})	3.0	Pre	5.4	10	0.896	0.023
	3.0	Pre	5.5	10	0.813	0.027
	3.0	Pre	5.6	10	0.729	0.028
	3.0	Pre	5.7	10	0.459	0.034
	3.0	Pre	5.8	10	0.2	0.041
	3.0	Pre	5.9	10	0.037	0.007
	3.0	Pre	9.0	10	0	0
	3.0	Post	4.5	10	1	0
	3.0	Post	5.4	10	0.841	0.026
	3.0	Post	5.5	10	0.745	0.024
	3.0	Post	5.6	10	0.62	0.024
	3.0	Post	5.7	10	0.305	0.031
	3.0	Post	5.8	10	0.082	0.02
	3.0	Post	5.9	10	0.012	0.002
	3.0	Post	9.0	10	0	0

Supplementary Table 4. **Statistical details from experiments shown in Fig. 1k-m. N = number of preparations included in the condition. For SL 2.4, a total of 10 trials were run from psoas collected from 4 mice (2 female / 2 male). For SL 2.7, a total of 10 trials were run from psoas collected from 6 mice (4 female / 2 male). For SL 3.0, a total of 10 trials were run from psoas collected from 4 mice (3 female / 1 male).**

Parameter	SL (μm)	Treatment	pCa	N	mean	SE	ANOVA	df 1	df 2	F stat	Prob > F
K_{tr}	2.4	Pre	4.5	10	1	0	Treatment	1	79	52.67	**$2.39x10^{-10}$**
	2.4	Pre	5.3	9	0.926	0.043					
	2.4	Pre	5.4	10	0.874	0.034	pCa	4	79	38.03	**$1.01x10^{-17}$**
	2.4	Pre	5.5	10	0.78	0.037					
	2.4	Pre	5.6	10	0.732	0.044	Interaction	4	79	3.36	**0.014**
	2.4	Pre	5.7	10	0.516	0.049					
	2.4	Post	4.5	10	1	0					
	2.4	Post	5.3	9	1.028	0.024					
	2.4	Post	5.4	10	0.986	0.026					
	2.4	Post	5.5	10	1.025	0.051					
	2.4	Post	5.6	10	1.007	0.059					
	2.4	Post	5.7	10	0.602	0.054					
K_{tr}	2.7	Pre	4.5	10	1	0	Treatment	1	77	39.84	**$1.6x10^{-8}$**
	2.7	Pre	5.4	10	0.913	0.024					
	2.7	Pre	5.5	10	0.898	0.024	pCa	4	75	122.53	**$4.2x10^{-32}$**
	2.7	Pre	5.6	**10**	0.882	0.045					
	2.7	Pre	5.7	9	0.669	0.045	Interaction	4	75	1.56	0.19
	2.7	Pre	5.8	10	0.317	0.03					
	2.7	Post	4.5	10	1	0					
	2.7	Post	5.4	9	1.034	0.026					
	2.7	Post	5.5	9	1.043	0.037					
	2.7	Post	5.6	9	1.065	0.044					
	2.7	Post	5.7	9	0.811	0.056					
	2.7	Post	5.8	9	0.366	0.03					
K_{tr}	3.0	Pre	4.5	10	1	0	Treatment	1	76	1.14	0.29
	3.0	Pre	5.4	10	0.907	0.03					
	3.0	Pre	5.5	10	0.891	0.03	pCa	4	76	57.84	**$2.2x10^{-22}$**
	3.0	Pre	5.6	10	0.843	0.036					
	3.0	Pre	5.7	10	0.683	0.03	Interaction	4	76	1.22	0.31
	3.0	Pre	5.8	9	0.39	0.031					
	3.0	Post	4.5	10	1	0					
	3.0	Post	5.4	10	0.955	0.038					
	3.0	Post	5.5	10	0.945	0.051					
	3.0	Post	5.6	10	0.936	0.048					
	3.0	Post	5.7	10	0.64	0.06					
	3.0	Post	5.8	6	0.351	0.056					

Bold values indicate significant effect. SE = standard error of the mean. T-tests are one sided.

Supplementary Table 5. **Statistical details from experiments shown in Supplementary Fig. 1f. N = number of preparations included in the condition. For SL 2.4, a total of 10 trials were run from psoas collected from 4 mice (2 female / 2 male). For SL 2.7, a total of 10 (9 post) trials were run from psoas collected from 6 mice (4 female / 2 male).**

Parameter	SL (μm)	Treatment	pCa	N	mean	SE
Tension ($mN\ mm^{-2}$)	2.4	Pre	4.5	10	27.691	4.542
	2.4	Pre	5.3	9	23.306	3.956
	2.4	Pre	5.4	10	24.791	3.919
	2.4	Pre	5.5	10	20.847	3.182
	2.4	Pre	5.6	10	16.786	2.656
	2.4	Pre	5.7	10	7.999	1.827
	2.4	Pre	5.8	9	1.137	0.566
	2.4	Pre	5.9	9	0.307	0.224
	2.4	Pre	9	10	0	0
	2.4	Post	4.5	10	26.463	4.498
	2.4	Post	5.3	9	22.562	4.229
	2.4	Post	5.4	10	22.735	3.957
	2.4	Post	5.5	10	18.875	3.653
	2.4	Post	5.6	10	12.086	2.544
	2.4	Post	5.7	10	4.007	1.115
	2.4	Post	5.8	9	0.429	0.232
	2.4	Post	5.9	9	0.063	0.043
	2.4	Post	9	10	0	0
Tension ($mN\ mm^{-2}$)	2.7	Pre	4.5	10	50.745	6.935
	2.7	Pre	5.4	10	45.267	6.637
	2.7	Pre	5.5	10	42.513	6.8
	2.7	Pre	5.6	10	36.495	5.897
	2.7	Pre	5.7	10	23.522	4.638
	2.7	Pre	5.8	10	8.696	3.803
	2.7	Pre	5.9	10	0.479	0.403
	2.7	Pre	9	10	0	0
	2.7	Post	4.5	9	57.541	8.391
	2.7	Post	5.4	9	51.427	6.015
	2.7	Post	5.5	9	47.222	7.157
	2.7	Post	5.6	9	37.691	4.601
	2.7	Post	5.7	9	23.491	3.812
	2.7	Post	5.8	9	5.616	1.366
	2.7	Post	5.9	9	0.284	0.184
	2.7	Post	9	9	0	0

SE = standard error of the mean

Supplementary Table 6. **Statistical details from experiments shown in Supplementary Fig. 1f. N = number of preparations included in the condition. For SL 3.0, a total of 10 trials were run from psoas collected from 4 mice (3 female / 1 male).**

Parameter	SL (µm)	Treatment	pCa	N	mean	SE
Tension	3.0	Pre	4.5	10	63.43	8.398
(mN mm^{-2})	3.0	Pre	5.4	10	51.526	6.728
	3.0	Pre	5.5	10	48.379	6.282
	3.0	Pre	5.6	10	40.702	5.153
	3.0	Pre	5.7	10	28.243	4.369
	3.0	Pre	5.8	10	13.543	3.484
	3.0	Pre	5.9	10	2.449	0.554
	3.0	Pre	9	10	0	0
	3.0	Post	4.5	10	60.728	7.196
	3.0	Post	5.4	10	49.661	7.013
	3.0	Post	5.5	10	44.657	6.068
	3.0	Post	5.6	10	36.164	5.185
	3.0	Post	5.7	10	19.582	3.733
	3.0	Post	5.8	10	5.951	1.65
	3.0	Post	5.9	10	0.757	0.152
	3.0	Post	9	10	0	0

SE = standard error of the mean

Supplementary Table 7. **Statistical details from experiments shown in Figure 1f and Supplementary Figure 1d. N = number of preparations included in the condition. A total of 9 trials were run from psoas collected from 2 mice for the pre condition (1 female, 1 male). A total of 10 trials were run from psoas collected from 3 mice for the post condition (1 female / 2 male).**

Parameter	SL (µm)	Treatment	pCa	N	mean	SE	ANOVA	df 1	df 2	F stat	Prob > F
Passive Tension	2.2	Pre	9	9	0.921	0.063	SL	4	68	316.98	**3.6×10^{-43}**
(mN mm^{-2})	2.4	Pre	9	9	2.383	0.281					
	2.6	Pre	9	9	3.75	0.339	Treatment	1	17	0.31	0.59
	2.8	Pre	9	9	7.717	1.04					
	3.0	Pre	9	9	10.274	1.695	Interaction	4	68	1.68	0.17
	2.2	Post	9	10	0.874	0.083					
	2.4	Post	9	10	1.822	0.178					
	2.6	Post	9	10	3.389	0.344					
	2.8	Post	9	10	6.635	0.795					
	3.0	Post	9	10	12.374	2.21					
Passive Tension	2.2	Pre	9	9	0.107	0.016	SL	3	51	527.67	**2.3×10^{-38}**
(mN mm^{-2})	2.4	Pre	9	9	0.277	0.043					
	2.6	Pre	9	9	0.409	0.039	Treatment	1	17	2.84	0.11
	2.8	Pre	9	9	0.805	0.067					
	3.0	Pre	9	9	1	0	Interaction	3	51	2.2	0.1
	2.2	Post	9	10	0.085	0.012					
	2.4	Post	9	10	0.183	0.028					
	2.6	Post	9	10	0.324	0.044					
	2.8	Post	9	10	0.599	0.049					
	3.0	Post	9	10	1	0					

Bold values indicate significant effect. SE = standard error of the mean

Supplementary Table 8. **Statistical details from experiments shown in Figure 1 k-m, and Supplementary Figure 1e. N = number of preparations included in the condition. For SL 2.4, a total of 10 trials were run from psoas collected from 4 mice (2 female, 2 male). For SL 2.7, a total of 10 (9 post) trials were run from psoas collected from 6 animals (4 female, 2 male). For SL 3.0, a total of 10 trials were run from psoas collected from 4 animals (3 female/ 1 male).**

Parameter	SL (μm)	Treatment	pCa	N	mean	SE	ANOVA	df 1	df 2	F stat	Prob > F
Maximal Tension	2.4	Pre	4.5	10	27.691	4.542	SL	2	27	8.60	**0.001**
(mN mm^{-2})	2.4	Post	4.5	10	26.463	4.498					
	2.7	Pre	4.5	10	50.745	6.688	Treatment	1	26	0.045	0.83
	2.7	Post	4.5	9	57.541	7.917					
	3.0	Pre	4.5	10	63.43	8.398	Interaction	2	26	3.802	**0.036**
	3.0	Post	4.5	10	60.728	7.196					
Ktr	2.4	Pre	4.5	10	11.248	0.862	Treatment	1	79	76.940	**$2.7x10^{-13}$**
(s^{-1})	2.4	Pre	5.3	9	10.283	0.986					
	2.4	Pre	5.4	10	9.788	0.842	pCa	4	79	51.661	**$2.6x10^{-21}$**
	2.4	Pre	5.5	10	8.714	0.68					
	2.4	Pre	5.6	10	8.16	0.687	Interaction	4	79	2.0062	0.10
	2.4	Pre	5.7	10	5.521	0.368					
	2.4	Post	4.5	10	11.808	0.762					
	2.4	Post	5.3	9	12.234	0.947					
	2.4	Post	5.4	10	11.621	0.764					
	2.4	Post	5.5	10	12.083	0.921					
	2.4	Post	5.6	10	11.711	0.762					
	2.4	Post	5.7	10	6.829	0.413					
Ktr	2.7	Pre	4.5	10	9.193	0.777	Treatment	1	75	22.42	**$1x10^{-5}$**
(s^{-1})	2.7	Pre	5.4	10	8.184	0.635					
	2.7	Pre	5.5	10	8.346	0.7	pCa	4	74	120.14	**$9.0x10^{-32}$**
	2.7	Pre	5.6	10	7.81	0.567					
	2.7	Pre	5.7	9	5.848	0.559	Interaction	4	74	0.46	0.77
	2.7	Pre	5.8	10	2.724	0.317					
	2.7	Post	4.5	9	9.516	0.583					
	2.7	Post	5.4	9	9.65	0.573					
	2.7	Post	5.5	9	9.939	0.67					
	2.7	Post	5.6	9	10.344	0.8					
	2.7	Post	5.7	9	8.228	1.088					
	2.7	Post	5.8	9	3.315	0.335					
Ktr	3.0	Pre	4.5	10	7.203	0.563	Treatment	1	76	4.27	**0.042**
(s^{-1})	3.0	Pre	5.4	10	6.483	0.517					
	3.0	Pre	5.5	10	6.419	0.554	pCa	4	76	92.60	**$1.9x10^{-28}$**
	3.0	Pre	5.6	10	6.118	0.632					
	3.0	Pre	5.7	10	4.905	0.43	Interaction	4	76	2.76	**0.034**
	3.0	Pre	5.8	9	2.751	0.23					
	3.0	Post	4.5	10	8.109	0.917					
	3.0	Post	5.4	10	7.53	0.691					
	3.0	Post	5.5	10	7.441	0.703					
	3.0	Post	5.6	10	7.442	0.73					
	3.0	Post	5.7	10	5.054	0.635					
	3.0	Post	5.8	6	2.639	0.316					

Bold values indicate significant effect. SE = standard error of the mean

Supplementary Table 9. **Statistical details from experiments shown in Figure 2. N = number of preparations included in the condition. A total of 37 trials were run from psoas collected from 15 mice (9 male / 6 female).**

Parameter	SL (µm)	Treatment	N	mean	SE	ANOVA	DF_1	DF_2	F stat	Prob > F
d_{10} (nm)	2.4	Pre	36	39.004	0.363	SL	2	162	58.12	**$5.4x10^{-33}$**
	2.4	Post	33	39.763	0.313	Treatment	1	163	14.90	**$9.9x10^{-10}$**
	2.7	Pre	33	37.912	0.441	Interaction	2	162	0.08	0.93
	2.7	Post	34	38.761	0.343					
	3.0	Pre	32	36.646	0.487					
	3.0	Post	34	37.361	0.426					
σ_D (nm^{-1})	2.4	Pre	21	7.817	0.253	SL	2	109	31.07	**$2.1x10^{-11}$**
	2.4	Post	20	8.103	0.304	Treatment	1	112	0.27	0.60
	2.7	Pre	18	9.226	0.426	Interaction	2	109	0.23	0.79
	2.7	Post	18	8.902	0.424					
	3.0	Pre	15	10.293	0.603					
	3.0	Post	21	10.616	0.524					
S_{T3} (nm)	2.4	Pre	33	12.644	0.007	SL	2	144	7.48	**$8.0x10^{-4}$**
	2.4	Post	29	12.631	0.007	Treatment	1	147	13.73	**$3.0x10^{-4}$**
	2.7	Pre	29	12.650	0.007	Interaction	2	144	0.03	0.97
	2.7	Post	31	12.638	0.009					
	3.0	Pre	29	12.659	0.007					
	3.0	Post	32	12.647	0.007					
S_{A6} (nm)	2.4	Pre	32	5.818	0.005	SL	2	134	54.02	**$6.3\ x10^{-18}$**
	2.4	Post	24	5.804	0.005	Treatment	1	136	36.81	**$1.2\ x10^{-8}$**
	2.7	Pre	30	5.841	0.005	Interaction	2	134	1.12	0.33
	2.7	Post	27	5.826	0.005					
	3.0	Pre	29	5.845	0.005					
	3.0	Post	29	5.835	0.005					
S_{A7} (nm)	2.4	Pre	28	5.062	0.003	SL	2	110	26.01	**$5.7\ x10^{-10}$**
	2.4	Post	23	5.046	0.004	Treatment	1	113	15.42	**$1.4\ x10^{-4}$**
	2.7	Pre	22	5.071	0.004	Interaction	2	109	2.35	0.10
	2.7	Post	20	5.060	0.004					
	3.0	Pre	26	5.076	0.003					
	3.0	Post	23	5.073	0.004					
S_{gActin} (nm)	2.4	Pre	28	2.708	0.002	SL	2	99	65.67	**$7.1x10^{-19}$**
	2.4	Post	20	2.699	0.002	Treatment	1	101	46.77	**$6.2x10^{-10}$**
	2.7	Pre	22	2.715	0.002	Interaction	2	99	3.62	**0.03**
	2.7	Post	17	2.709	0.002					
	3.0	Pre	24	2.718	0.002					
	3.0	Post	22	2.715	0.002					

Bold values indicate significant effect. SE = standard error of the mean

Supplementary Table 10. **Statistical details from experiments shown in Figure 3. N = number of preparations included in the condition. A total of 37 trials were run from psoas collected from 15 mice (9 male /6 female).**

Parameter	SL (µm)	Treatment	N	mean	SE	ANOVA	DF_1	DF_2	F stat	Prob > F
$I_{M2\ cluster}$	2.4	Pre	28	0.371	0.036	SL	2	129	4.05	**0.02**
	2.4	Post	28	0.206	0.022	Treatment	1	135	26.60	**8.7×10^{-7}**
	2.7	Pre	24	0.292	0.035	Interaction	2	129	4.91	**0.01**
	2.7	Post	27	0.216	0.019					
	3.0	Pre	28	0.242	0.021					
	3.0	Post	28	0.211	0.022					
I_{11}/I_{10}	2.4	Pre	35	0.636	0.029	SL	2	157	4.67	**0.01**
	2.4	Post	31	0.749	0.028	Treatment	1	159	19.42	**3.0×10^{-8}**
	2.7	Pre	32	0.726	0.042	Interaction	2	157	0.35	0.71
	2.7	Post	34	0.827	0.035					
	3.0	Pre	30	0.708	0.043					
	3.0	Post	33	0.860	0.053					
S_{M3} (nm)	2.4	Pre	32	14.184	0.008	SL	2	146	36.34	**1.6×10^{-13}**
	2.4	Post	28	14.223	0.007	Treatment	1	147	18.24	**3.0×10^{-5}**
	2.7	Pre	31	14.231	0.009	Interaction	2	143	2.39	**0.09**
	2.7	Post	31	14.264	0.009					
	3.0	Pre	31	14.259	0.009					
	3.0	Post	31	14.266	0.010					
S_{M6} (nm)	2.4	Pre	31	7.163	0.002	SL	2	134	30.07	**1.6×10^{-11}**
	2.4	Post	26	7.169	0.002	Treatment	1	137	12.75	**5.0×10^{-4}**
	2.7	Pre	29	7.171	0.002	Interaction	2	134	0.12	0.89
	2.7	Post	29	7.176	0.003					
	3.0	Pre	28	7.176	0.003					
	3.0	Post	27	7.180	0.003					

Bold values indicate significant effect. SE = standard error of the mean

Supplementary Table 11. **Statistical details from experiments shown in Supplementary Figure 2 (controls). N = number of preparations included in the condition. A total of 20 trials were run from psoas collected from 6 mice (3 male / 3 female).**

Parameter	SL (µm)	Treatment	N	mean	SE	ANOVA	DF_1	DF_2	F stat	Prob > F
d_{10} (nm)	2.4	Pre	19	39.54	0.32	Treatment	1	37	0.79	0.40
	2.4	Post	8	39.34	0.46	SL	1	33	46.08	**1.0×10^{-7}**
	2.7	Pre	19	37.40	0.44	Interaction	1	33	0.04	0.84
	2.7	Post	8	37.32	0.62					
I_{11}/I_{10}	2.4	Pre	19	0.504	0.049	Treatment	1	37	0.18	0.68
	2.4	Post	8	0.501	0.073	SL	1	32	10.40	**0.003**
	2.7	Pre	19	0.634	0.058	Interaction	1	32	0.04	0.84
	2.7	Post	8	0.642	0.088					
σ_D (nm^{-1})	2.4	Pre	17	98.854	0.214	Treatment	1	45	0.24	0.62
	2.4	Post	7	8.793	0.284	SL	1	28	21.35	**7.8×10^{-5}**
	2.7	Pre	17	10.098	0.236	Interaction	1	26	0.00	0.95
	2.7	Post	8	9.996	0.276					
S_{M3} (nm)	2.4	Pre	17	14.185	0.015	Treatment	1	37	0.02	0.88
	2.4	Post	10	14.202	0.022	SL	1	33	29.94	**4.6×10^{-6}**
	2.7	Pre	19	14.247	0.015	Interaction	1	33	0.08	0.77
	2.7	Post	10	14.271	0.022					
S_{M6} (nm)	2.4	Pre	13	7.150	0.005	Treatment	1	21	0.37	0.55
	2.4	Post	4	7.152	0.010	SL	1	17	20.43	**3.0×10^{-4}**
	2.7	Pre	13	7.161	0.081	Interaction	1	17	0.18	0.67
	2.7	Post	4	7.179	0.016					
S_{T3} (nm)	2.4	Pre	16	12.608	0.012	Treatment	1	32	0.30	0.59
	2.4	Post	8	12.617	0.021	SL	1	26	26.98	**2.0×10^{-5}**
	2.7	Pre	16	12.652	0.010	Interaction	1	26	0.01	0.91
	2.7	Post	7	12.671	0.021					
S_{A6} (nm)	2.4	Pre	15	5.808	0.008	Treatment	1	28	1.46	0.24
	2.4	Post	8	5.817	0.010	SL	1	26	55.07	**7.0×10^{-8}**
	2.7	Pre	16	5.847	0.011	Interaction	1	26	0.22	0.64
	2.7	Post	8	5.856	0.011					
S_{A7} (nm)	2.4	Pre	6	5.060	0.007	Treatment	1	17	0.16	0.70
	2.4	Post	5	5.063	0.010	SL	1	11	6.24	**0.03**
	2.7	Pre	8	5.079	0.011	Interaction	1	10	0.12	0.74
	2.7	Post	3	5.073	0.011					
S_{gActin} (nm)	2.4	Pre	6	2.707	0.006	Treatment	1	14	0.56	0.46
	2.4	Post	5	2.708	0.006	SL	1	9	10.59	**0.009**
	2.7	Pre	8	2.719	0.005	Interaction	1	9	0.10	0.76
	2.7	Post	3	2.719	0.006					

Bold values indicate significant effect. SE = standard error of the mean

Supplementary Table 12. **Statistical details between control and SnoopC2 X-ray study datasets. Connecting letters report = matching letters between conditions are not significantly different.**

Parameter	ANOVA	DF_1	DF_2	F stat	Prob > F	Study	SL (μm)	Connecting Letters
d_{10} (nm)	Study	1	53	0.0002	0.99	Control	2.4	A, B
	SL	1	50	46.08	**1.0x10^{-8}**	TEV	2.4	A, C
	Interaction	1	50	0.04	**0.02**	Control	2.7	C, D
						TEV	2.7	B, D
I_{11}/I_{10}	Study	1	51	3.23	0.08	Control	2.4	A
	SL	1	49	16.73	**1.6x10^{-4}**	TEV	2.4	A
	Interaction	1	49	0.19	0.66	Control	2.7	B
						TEV	2.7	B
σ_D (nm^{-1})	Study	1	37	7.96	**0.01**	Control	2.4	A
	SL	1	35	28.70	**5.4x10^{-6}**	TEV	2.4	B
	Interaction	1	35	0.08	0.78	Control	2.7	C
						TEV	2.7	D
S_{M3} (nm)	Study	1	48	0.87	0.36	Control	2.4	A
	SL	1	43	39.67	**1.3x10^{-7}**	TEV	2.4	A
	Interaction	1	43	0.44	0.51	Control	2.7	B
						TEV	2.7	B
S_{M6} (nm)	Study	1	45	0.18	0.67	Control	2.4	A
	SL	1	38	61.58	**1.8 x10^{-9}**	TEV	2.4	A
	Interaction	1	38	16.40	**2.0x10^{-4}**	Control	2.7	B
						TEV	2.7	B
S_{T3} (nm)	Study	1	46	1.81	0.18	Control	2.4	A
	SL	1	41	20.01	**6.0x10^{-5}**	TEV	2.4	B
	Interaction	1	41	11.49	**0.002**	Control	2.7	B
						TEV	2.7	B
S_{A6} (nm)	Study	1	46	0.01	0.92	Control	2.4	A
	SL	1	42	75.60	**6.8x10^{-11}**	TEV	2.4	A
	Interaction	1	42	3.55	0.07	Control	2.7	B
	Study					TEV	2.7	B
S_{A7} (nm)	Study	1	37	0.21	0.65	Control	2.4	A
	SL	1	29	16.31	**4.0x10^{-4}**	TEV	2.4	A
	Interaction	1	29	0.27	0.61	Control	2.7	B
						TEV	2.7	B
S_{gActin} (nm)	Study	1	37	0.20	0.66	Control	2.4	A
	SL	1	26	49.81	**1.7x10^{-7}**	TEV	2.4	A
	Interaction	1	26	0.12	0.74	Control	2.7	B
						TEV	2.7	B

Bold values indicate significant effect. SE = standard error of the mean
