# Supplementary material for: Myosin-binding protein C regulates the sarcomere lattice and stabilizes the OFF states of myosin heads
Source: Nat Commun. 2024 Mar 23;15:2628. doi: 10.1038/s41467-024-46957-7 (PMC10960836; doi:10.1038/s41467-024-46957-7)
Supplement: Supplementary file 3 — Reporting Summary [file 41467_2024_46957_MOESM3_ESM.pdf]

nature portfolio

Corresponding author(s): Anthony Hessel, PhD
Samantha Harris, PhD

Last updated by author(s): Feb 27, 2024

Reporting Summary

Nature Portfolio wishes to improve the reproducibility of the work that we publish. This form provides structure for consistency and transparency in reporting. For further information on Nature Portfolio policies, see our Editorial Policies and the Editorial Policy Checklist.

Statistics

For all statistical analyses, confirm that the following items are present in the figure legend, table legend, main text, or Methods section.

n/a	Confirmed	
☐	☒	The exact sample size (n) for each experimental group/condition, given as a discrete number and unit of measurement
☐	☒	A statement on whether measurements were taken from distinct samples or whether the same sample was measured repeatedly
☐	☒	The statistical test(s) used AND whether they are one- or two-sided<br>*Only common tests should be described solely by name; describe more complex techniques in the Methods section.*
☐	☒	A description of all covariates tested
☐	☒	A description of any assumptions or corrections, such as tests of normality and adjustment for multiple comparisons
☐	☒	A full description of the statistical parameters including central tendency (e.g. means) or other basic estimates (e.g. regression coefficient) AND variation (e.g. standard deviation) or associated estimates of uncertainty (e.g. confidence intervals)
☐	☒	For null hypothesis testing, the test statistic (e.g. F, t, r) with confidence intervals, effect sizes, degrees of freedom and P value noted<br>*Give P values as exact values whenever suitable.*
☒	☐	For Bayesian analysis, information on the choice of priors and Markov chain Monte Carlo settings
☒	☐	For hierarchical and complex designs, identification of the appropriate level for tests and full reporting of outcomes
☒	☐	Estimates of effect sizes (e.g. Cohen's d, Pearson's r), indicating how they were calculated

Our web collection on statistics for biologists contains articles on many of the points above.

Software and code

Policy information about availability of computer code

Data collection	Mechanical data collected with the 600A: Real-time muscle acquitisiton and analysis system (Aurora Scientific).
Data analysis	Mechanical data analyzed with the 600A: Real-time muscle acquitisiton and analysis system (Aurora Scientific). X-ray diffraction data analyzed with MuscleX V.1.22 (BioCAT Laboratory; https://musclex.readthedocs.io/).

For manuscripts utilizing custom algorithms or software that are central to the research but not yet described in published literature, software must be made available to editors and reviewers. We strongly encourage code deposition in a community repository (e.g. GitHub). See the Nature Portfolio guidelines for submitting code & software for further information.

Data

Policy information about availability of data

All manuscripts must include a data availability statement. This statement should provide the following information, where applicable:

- Accession codes, unique identifiers, or web links for publicly available datasets
- A description of any restrictions on data availability
- For clinical datasets or third party data, please ensure that the statement adheres to our policy

All data are available in the main text or the supplementary materials, or available upon reasonable request.

Research involving human participants, their data, or biological material

Policy information about studies with human participants or human data. See also policy information about sex, gender (identity/presentation), and sexual orientation and race, ethnicity and racism.

Reporting on sex and gender	N/A
Reporting on race, ethnicity, or other socially relevant groupings	N/A
Population characteristics	N/A
Recruitment	N/A
Ethics oversight	N/A

Note that full information on the approval of the study protocol must also be provided in the manuscript.

Field-specific reporting

Please select the one below that is the best fit for your research. If you are not sure, read the appropriate sections before making your selection.

[x] Life sciences [ ] Behavioural & social sciences [ ] Ecological, evolutionary & environmental sciences

For a reference copy of the document with all sections, see nature.com/documents/nr-reporting-summary-flat.pdf

Life sciences study design

All studies must disclose on these points even when the disclosure is negative.

Sample size	Sample size was determined using the freeware G*Power (https://www.psychologie.hhu.de/arbeitsgruppen/allgemeine-psychologie-und-arbeitspsychologie/gpower) and JMP (V.14; SAS Institute), and using past similar experiments to estimate effect size and physiologically relevant changes.
Data exclusions	For X-ray diffraction intensities, if data was not fittable by a guassian curve, we excluded it, as typically done. No other data were excluded from our analysis. For mechanics, experiments were excluded if maximal force generated at the end of the experiment had decreased over 25% from the initial maximal force measurement (rundown).
Replication	We did not take measures to verify the reproducibility of the experimental findings. The time pressure at X-ray facilities makes proper replication experiments difficult to schedule. However, the data were collected over multiple days, and no inter-day variability was noted.
Randomization	Within each colony, mouse were radomly selected from the colony for use in this study. Each samples was evaluated in each experimental condition, and an individual random effect was added to statistical models to account for this repeated measurement.
Blinding	Investigators were not blinded during data collection because X-ray data collection for each study period used only used type of mouse. For logistical reasons, we could not change this project feature. Investigators were blind during the initial analysis of X-ray diffraction datasets, but for data handling reasons (hundreds of large files per sample) it was not possible to blind the investigator during quality assurance.

Reporting for specific materials, systems and methods

We require information from authors about some types of materials, experimental systems and methods used in many studies. Here, indicate whether each material, system or method listed is relevant to your study. If you are not sure if a list item applies to your research, read the appropriate section before selecting a response.

Materials & experimental systems

n/a	Involved in the study	
[ ]	[x]	Antibodies
[x]	[ ]	Eukaryotic cell lines
[x]	[ ]	Palaeontology and archaeology
[ ]	[x]	Animals and other organisms
[x]	[ ]	Clinical data
[x]	[ ]	Dual use research of concern
[x]	[ ]	Plants

Methods

n/a	Involved in the study	
[x]	[ ]	ChIP-seq
[x]	[ ]	Flow cytometry
[x]	[ ]	MRI-based neuroimaging

Antibodies

Antibodies used	Primary Antibodies:<br>1. MYBPC2 (fMyBP-C) Polyclonal Antibody. Supplied by ThermoFisher. Catalog #: PA5-83638. Host/Isotope Rabbit/IgG. Lot# XK3738509C<br>2. MYBPC1 antibody - BSA Free. Supplied by Novus Biologicals. Catalog #: NBP2-41157. Polyclonal Antibody. Host/Isotope: Rabbit/IgG. Lot # 6679-1404.<br>3. Actin Monoclonal Antibody (ACTN05 (C4)). Supplied by ThermoFisher. Catalog #: MA5-11869. Host/Isotope: Mouse/IgG1, kappa. Lot: XB3499085<br>Secondary Antibodies:<br>1. IRDye® 800CW Goat anti-Rabbit IgG Secondary Antibody. Supplied by LI-COR. Catalog # 926-32211. Lot #: 91030-13<br>2. IRDye® 680RD Goat anti-Mouse IgG Secondary Antibody. Supplied by LI-COR. Catalog #: 926-68070. Lot #: D10901-15.
Validation	Primary Antibodies:<br>1. MYBPC2 Antibody PA5-83638. Immunogen sequence: FKGKWLELGS KSGARFSFKE SHNSASNVYT VELHIGKVVL GDRGYYRLEV KAKDTCDSCG FNIDVEAPRQ DASGQSLESF KRTSEKKSDT A "Relative expression in different tissues in IHC: Detection of differential expression levels of MYBPC2 demonstrates antibody specificity. Immunohistochemical analysis of MYBPC2 using anti-MYBPC2 Polyclonal Antibody (Product #PA5-83638), shows significant staining of MYBPC2 in skeletal muscle and shows minimal or weak staining in heart muscle tissues. The relative expression levels of MYBPC2 within each tissue is shown using RNA-Seq." This antibody is currently validated to target human fMyBP-C and has not officially been validated in mouse outside of our ability to identify the "cut" protein as shown in Figure 1.<br>2. MYBPC1 antibody NBP2-41157. Antibody was raised against an 18 amino acid synthetic peptide near the amino terminus of human MYBPC1. The immunogen is located within amino acids 160 - 210 of MYBPC1. Amino Acid Squence: DIRSAFKRSGEGQEDAGE.At least five isoforms of MYBPC1 are known to exist. MYBPC1 antibody is predicted not to cross-react with other MYBPC family members.<br>3. Actin Monoclonal Antibody MA5-11869: Species Reactivity: Bovine, Dog, Chicken, Human, Mouse, Pig, Protozoa, Rabbit, Rat. Published species: C. elegans, Chordate, Dog, Ferret, Fish, Fruit fly, Hamster, Human, Insect, Mouse, Non-human primate, Pig, Rabbit, Rat, Rhesus monkey, Xenopus, Yeast, Zebrafish. "Western blot analysis of actin was performed by loading 25 µg of various whole cell lysates and 10 µL of PageRuler Plus Prestained Protein Ladder (Product # 26619) per well onto a 4-20% Tris-HCl polyacrylamide gel. Proteins were transferred to a PVDF membrane and blocked with 5% BSA in TBST for at least 1 hour. The membrane was probed with a pan actin monoclonal antibody (Product # MA5-11869) at a dilution of 1:3200 overnight at 4°C on a rocking platform, washed in TBS-0.1%Tween-20, and probed with HRP-conjugated goat anti-mouse IgG secondary antibody (Product # 31430) at a dilution of 1:40,000 for at least 1 hour. Chemiluminescent detection was performed using SuperSignal West Pico (Product # 34080)."<br>Secondary Antibodies:<br>IRDye® 800CW Goat anti-Rabbit IgG Secondary Antibody 926-32211: Isolation of specific antibodies was accomplished by affinity chromatography using pooled rabbit IgG covalently linked to agarose. Based on ELISA and flow cytometry, this antibody reacts with the heavy and light chains of rabbit IgG, and with the light chains of rabbit IgM and IgA. This antibody was tested by dot blot and and/or solid-phase adsorbed for minimal cross-reactivity with human, mouse, rat, sheep, and chicken serum proteins, but may cross-react with immunoglobulins from other species. The conjugate has been specifically tested and qualified for Western blot and In-Cell Western™ Assay applications.<br>IRDye® 680RD Goat anti-Mouse IgG Secondary Antibody 926-68070. Isolation of specific antibodies was accomplished by affinity chromatography using pooled mouse IgG covalently linked to agarose. Based on ELISA and flow cytometry, this antibody reacts with the heavy and light chains of mouse IgG1, IgG2a, IgG2b, and IgG3, and with the light chains of mouse IgM and IgA. This antibody was tested by dot blot and and/or solid-phase adsorbed for minimal cross-reactivity with human, rabbit, goat, rat, and horse serum proteins, but may cross-react with immunoglobulins from other species. The conjugate has been specifically tested and qualified for Western blot and In-Cell Western™ Assay applications.

Animals and other research organisms

Policy information about studies involving animals; ARRIVE guidelines recommended for reporting animal research, and Sex and Gender in Research

Laboratory animals	Genetically homozygous and wildtype adult SNOOPC2 (Background: C57BL6/NJ) mice (age range, 2 – 6 months). Mice are housed in a sterile facility with a 14 hour light / 10 hour dark cycle, where the lights are turned on from 5am to 7pm. The temperature of the room is kept around 72°F. The humidity of the mouse facility typically ranges between 40-60% humidity. For mechanics experiments, 10 SNOOPC2 mice were used, four of which were male and six of which were female. From these 10 animals, 49 muscle fibers were used through the entirety of the mechanics dataset. For X-ray studies, 15 homozygous SNOOPC2 mice were selected, 9 of which were male and 6 of which were female. For X-ray controls, 6 wild type SNOOPC2 mice were selected. 3 of which were male and 3 of which were female.
Wild animals	The study did not provide wild animals
Reporting on sex	Both male and female mice were used in this study design, as we have previously found no sex-based differences in TEV protease treatment effect in skeletal fibers (Hessel et al. 2022 PNAS).
Field-collected samples	The study did not involve field-collected samples.
Ethics oversight	Animal procedures were approved and performed according to the guidelines of the local animal care and use committee (IACUC) of the University of Arizona. IACUC Protocol # 13-446

Note that full information on the approval of the study protocol must also be provided in the manuscript.
